# Thrombosis with Behçet's disease should be evaluated different conditions

**DOI:** 10.11604/pamj.2015.20.117.3571

**Published:** 2015-02-10

**Authors:** Ilknur Balta, Sevket Balta, Mustafa Demir, Cengiz Ozturk, Sait Demirkol

**Affiliations:** 1 Department of Dermatology, Kecioren Training and Research Hospital, Ankara, Turkey; 2 Department of Cardiology, Eskisehir Military Hospital, Eskisehir, Turkey; 3 Department of Cardiology, Gulhane Medical Academy, Ankara, Turkey

**Keywords:** Thrombosis, Behçet′s disease, massive pulmonary

## Opinion

Dear Editor,

We read the article ‘‘Intracardiac thrombosis in Behçet's disease: a rare complication’ by Ghita Saghi et al with interest [1]. The authors reported a case with mobile thrombus in the right ventricle and a massive pulmonary embolism by a magnetic resonance Imaging scan (MRI), which was successfully treated conservatively.

Vascular involvement and thrombotic tendency is a potentially lifethreatening condition in patients with Behçet′s disease. Deep vein thrombosis and pulmonary embolism are the most common manifestation but other locations may also occur. A risk factor of thrombosis was observed in many patients. Most of patients have commonly a tendency to be thrombophilias including hereditary thrombophilia, an antiphospholipids syndrome, Behçet′s disease and neoplasia. Medical therapy and surgical resection can be mentioned these thrombotic complication. Surgical treatment could be considered because the right atrial thrombus was mobile and it caused a massive pulmonary embolism.

We previously reported case of a 48-year-old male patient with Behçet's disease who presented with right heart thrombus dissolved after medical management [2]. In this case, because many conditions could cause thrombotic events [3], we investigated the hypercoagulability tendeny conditions including the higher protein C, protein S, homocystine and positive antiphospholipid antibodies. We also analysed the prothrombin gene and Factor V Leiden mutation. Because differential diagnosis for thrombosis is an important for these conditions, the authors had mentioned these factors.

Furthermore, in a previous case report, the authors reported a case with diffuse thrombosis extending from vena cava superior to brachiocephalic vein accompanied by dural sinus thrombosis, which was successfully treated with oral anticoagulant and thrombolytic therapy. After warfarin therapy a significant drop in hemoglobin level occurred. The anticoagulant therapy was stopped due to suspected GIS hemorrhage and antiaggregant therapy was replaced. In this context, thrombolytic and warfarin treatment should not be withheld if necessary, because they may lead to serious complications [4]. As a conclusion, a case with with mobile thrombus in the right ventricle and a massive pulmonary embolism by a magnetic resonance Imaging scan (MRI), which was successfully treated conservatively as presented in current case report. However, thrombotic events may be associated very different conditions and warfarin treatment may cause serious complications and the pivotal roles of those factors evaluate further reports.
